# Efficient Pattern Modeling Method for Parabolic Cylindrical Antennas Incorporating Multi-Source Structural Errors

**DOI:** 10.3390/s26030933

**Published:** 2026-02-01

**Authors:** Shiyue Xue, Weibin Liang, Mingming Zhu, Shijie Ren

**Affiliations:** 1 Aerospace Information Research Institute, Chinese Academy of Sciences, Beijing 100094, China; 2 Key Laboratory of Target Cognition and Application Technology, Chinese Academy of Sciences, Beijing 100190, China; 3 School of Electronic, Electrical and Communication Engineering, University of Chinese Academy of Sciences, Beijing 100049, China; 4 Jiuquan Satellite Launch Center, Jiuquan 735400, China; 5 School of Information Science & Engineering, Yunnan University, Kunming 650500, China

**Keywords:** parabolic cylindrical antenna, pattern model, structural errors, Physical Optics, Fresnel approximation, Monte Carlo simulation

## Abstract

Parabolic cylindrical antennas are characterized by their structural simplicity, high radiation efficiency, and low manufacturing costs. Consequently, they are widely used in Earth observation and serve as a viable option for spaceborne Synthetic Aperture Radar (SAR) systems. However, structural errors in the phased array feed and the parabolic cylindrical reflector are inevitable during manufacturing, assembly, and operation. These errors significantly degrade the accuracy of antenna pattern models. To address this issue, this paper proposes a comprehensive radiation pattern model that accounts for structural errors in both the linear feed and the reflector. This approach enables precise pattern prediction and efficient in-orbit calibration. Specifically, the reflected far-field pattern is first calculated using the field superposition principle and the Physical Optics (PO) method. Specifically, the combined phase effects resulting from feed and reflector structural errors are superimposed to establish a direct integration pattern model for the parabolic cylindrical antenna. Given the high computational complexity of the direct integration model, a simplified model based on Fresnel approximation is proposed. This approach significantly reduces integration complexity while preserving the quadratic phase characteristics of the main lobe, thereby substantially improving computational efficiency. Simulation results verify that the simplified model maintains high accuracy in both normalized amplitude and phase. Furthermore, a partitioned calibration method is proposed to compensate for the absolute gain deviation inherent in the simplified model. By integrating weighting relationships derived from sensitivity analysis of individual errors, an empirical parameter is defined to quantify the correlation between total structural errors, antenna performance, and the prediction accuracy of the simplified model. The results indicate that reflector structural errors are the dominant factor affecting the overall performance of the antenna. In contrast, the prediction accuracy of the simplified model is found to be more sensitive to feed structural errors. The simplified model exhibits tolerance to structural errors far exceeding the wavelength, enabling it to effectively replace the direct integration model. This work provides new theoretical foundations and technical methods for tolerance design, performance assurance, in-orbit testing, and calibration of parabolic cylindrical antennas.

## 1. Introduction

Antennas in Synthetic Aperture Radar (SAR) primarily perform signal transmission and echo reception. They directly affect the imaging quality through patterns, polarization characteristics, and other factors, playing a key role in the design of SAR systems [1]. Large-aperture reflector antennas can collect more radar signals, enhancing image resolution and improving target detail and imaging quality. They are commonly used antenna forms in spaceborne SAR. Reflector antennas include rotating parabolic antennas, parabolic cylindrical antennas, stacked multi-beam parabolic antennas, and Cassegrain antennas. Among them, parabolic cylindrical antennas combine the advantages of the flexible beam scanning of a linear phased-array feed with the focusing characteristics of a parabolic cylindrical surface. They use phased array feed to excite the reflector, achieving efficient energy focusing and directional radiation, thereby enabling beam control [2]. They can meet the requirements of a wide frequency band and high gain [3]. Therefore, they have been widely applied. The AIRSAT Technology Group successfully developed the AIRSAT 01 and 02 satellites. As the first satellites in the AIRSAT constellation, these Ku-band SAR satellites used parabolic-cylindrical reflector SAR antennas, achieving a resolution better than 1 m. This marks the first application of parabolic-cylindrical antenna technology in China’s commercial satellite sector. China’s first marine salinity detection satellite is equipped with a new type of multi-band active- and passive-combined payload, MICAP. It also uses a combined active and passive multi-frequency detection system with a parabolic cylindrical antenna. While achieving high spatial resolution, it reduces system complexity and achieves detection accuracy of up to 0.1‰, reaching an internationally leading level [4,5].

In practical applications of parabolic cylindrical antennas, unavoidable structural errors introduce phase distortions and shifts in the energy distribution in the pattern. These effects influence key parameters, including main-lobe energy, beam pointing accuracy, sidelobe levels, and beamwidth. Ultimately, such changes degrade the overall far-field performance of the antenna [6]. Current studies on the impact of errors on antenna radiation characteristics mainly focus on either phased array feed error [7,8,9,10,11,12] or reflector structural errors [6,13,14], as well as the interaction between feed and reflector [15,16], which can not comprehensively reflect the actual application conditions of antennas. This study aims to develop an antenna pattern model by conducting a comprehensive analysis of feed and reflector errors, thereby enhancing the accuracy of pattern performance prediction.

Currently, various methods are used for approximate calculation of far-field pattern, including Geometric Optics (GO) [6], Physical Optics (PO) [15,17,18], diffraction method, and the Method of Moments (MoM) [15,18,19,20]. Comprehensive research shows that the essence of the PO method is approximating the Stratton–Chu integral equation under high-frequency conditions. During the modeling process, it can accurately capture the influence of shape, size, and surface errors on radiation characteristics and is often used for scattering calculations of electrically large, high-frequency objects [21,22]. Compared with other methods, the PO method adopts the principle of high-frequency localization. Even if the mutual coupling effect between elements is ignored [23], it still has higher accuracy. It better balances the computational complexity and pattern accuracy when analyzing the radiation characteristics of reflector antennas. Consequently, it has become the mainstream high frequency method for the analysis of antenna radiation characteristics. Current approaches for analyzing the electrical performance of parabolic cylindrical antennas primarily fall into two categories: analytical approximation techniques, such as the Aperture Integration Method (AIM) [24,25] and full-wave numerical methods (including the Method of Moments (MoM) and the Multilevel Fast Multipole Method (MLFMM) [26,27]). However, analytical approximation methods often overlook the discontinuity of discrete phased array feeds and the radiation patterns of individual elements. These methods struggle to accurately characterize the three-dimensional structural errors within the feed array. Conversely, although full-wave numerical methods achieve high accuracy, they are computationally prohibitive when applied to electrically large antennas. Therefore, there is a pressing need to develop a radiation pattern modeling method for parabolic cylindrical antennas that balances computational accuracy with efficiency, while comprehensively accounting for multi-source structural errors.

We designed a linear array-fed parabolic cylindrical antenna system comprising a linear phased array feed and a parabolic cylindrical reflector. Furthermore, a modeling method is proposed to accurately and efficiently quantify the influence of multi-source structural errors on the far-field radiation pattern. The main contributions of this work are summarized as follows:

1. A direct integration model is established to accurately quantify the combined effects of feed and reflector structural errors on far-field radiation characteristics.

2. An efficient calculation method based on the Fresnel approximation is proposed to address computational complexity.

3. The accuracy of the simplified model regarding beam shape and absolute gain is validated, and a partitioned compensation strategy is designed to correct absolute gain deviations.

4. The validity limits of the simplified model are defined, providing a reliable theoretical basis for evaluating the performance of parabolic cylindrical antennas in the presence of multiple error sources.

## 2. Direct Integration Pattern Modeling Considering the Feed and Reflector Structural Errors

### 2.1. Linear Phased Array Feed Pattern Model

The phased array feed achieves flexible control of beam direction and shape by adjusting the phase and amplitude of each TR module, enabling rapid beam scanning, beam reshaping, and precise spatial pointing [28,29,30]. According to the theory of phased array feeds, phased array antennas adjust the phase shifters to change the relative phase between elements. This adjustment steers the beam’s maximum radiation direction. As a result, the antenna achieves regular spatial scanning and predictable patterns [31]. The characteristics of the feed radiation field are determined by parameters such as the precise geometric dimensions of the feeding elements, the pattern, and the complex excitation coefficients. Based on the field superposition principle, the far-field strength of the phased array feed in any given direction is determined by the coherent superposition of the radiated fields from individual elements. This process involves a vector summation that accounts for both amplitude and phase. When the polarization directions of the elements are consistent, the vector sum can be simplified to scalar addition [32], thereby directly characterizing the radiation properties of the phased array feed.

**Figure 1:**
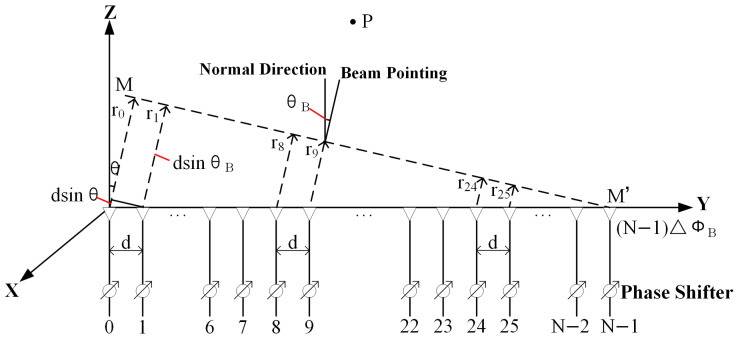
Linear phased array feed structure.

Based on the principles of phase scanning and the field superposition, the feed excitation model of a linear phased array feed consisting of *N* elements is analyzed (Figure 1). Assuming each feed is a point radiation source with equal-amplitude, phase-excited excitation without error. Assuming no mutual coupling between feeding elements, and that the spacing between each element is equal to *d*.

The far-field strength ${\vec{E}}_{n}$ at observation point *P* due to the *n*-th feeding element is given by the following:

(1) $${\vec{E}}_{n}=K_{n}\;\cdot \;\frac{e^{-jk{\vec{r}}_{n}}}{4\pi {\vec{r}}_{n}}\;\cdot \;I_{n}\;\cdot \;F_{e}\left(\theta ,\varphi \right)$$

where $K_{n}$ is a proportionality coefficient related to the radiation strength of the *n*-th element (identical for all elements with the same geometry [32]), $k=\frac{2\pi }{\lambda }$ is the wavenumber, λ is the wavelength, $I_{n}$ is the complex excitation current of the *n*-th element, and $F_{e}\left(\theta ,\varphi \right)$ is the element pattern that is determined solely by its geometry, dimensions, and independent of the array configuration. In modeling, the linear feed employs $F_{e}\left(\psi \right)\approx {\left(\cos\;\psi \right)}^{q}$ model to approximate the unit pattern function, where ψ represents the angle between the line-of-sight direction from the feed unit to a point on the reflector surface, and the feed’s main radiation direction, and *q* is the beamwidth factor.

When the observation point *P* is in the far-field, the distances from each element to the point can be approximately parallel. Taking the first element as the phase reference, the spatial phase difference caused by the different positions of each element in the ideal state is as follows:

(2) $$\Delta \phi_{n}=k\;\Delta {\vec{r}}_{n}=\begin{array}{c} kd\sin\;\theta \sin\;\varphi \end{array}$$

The overall directional pattern model of the linear phased array feed is synthesized based on the principle of electric field superposition as follows [33]:

(3) $$F\left(\theta ,\varphi \right)=\sum_{n=0}^{N-1}\begin{array}{c} F_{e}\left(\psi_{n}\right) \end{array}I_{n}e^{jn\Delta \phi_{n}}=\sum_{n=0}^{N-1}\begin{array}{c} F_{e}\left(\psi_{n}\right) \end{array}A_{n}e^{jn\varphi_{n}}\begin{array}{c} e^{jn\Delta \phi_{n}} \end{array}$$

where Δ$\phi_{n}$ is the spatial phase difference determined by relative element positions (sensitive to manufacturing errors, positional offsets, structural deformations), and $\varphi_{n}$ is the phase difference within the array controlled by two adjacent phase shifters (affected by errors such as source instability, channel distortion, phase quantization, mutual coupling).

In order to achieve rapid spatial scanning of the beam, a variable phase shifter is connected after each element [31], as shown in Figure 1, set the phase shift amounts of each element phase shifter to be respectively $0,\;\Delta \phi_{B},2\;\Delta \phi_{B},\ldots ,\left(N-1\right)\;\Delta \phi_{B}$. When the beam is steered toward $\left(\theta_{B},\varphi_{B}\right)$, the phase shifters are adjusted so that their phase shifts exactly compensate for the path-induced phase differences, resulting in coherent addition of the fields and a maximum in the desired direction. The phase difference within the array controlled by the phase shifter is $\varphi_{n}=\;\Delta \phi_{B}=\begin{array}{c} -kd\sin\;\theta_{B}\sin\;\varphi_{B} \end{array}$. By adjusting the phase difference within the array, the antenna beam direction is controlled. The beam direction changes from the direction of the array normal $\left(\theta =0,\varphi =0\right)$ to the direction of the beam $\theta_{B},\varphi_{B}$. The electromagnetic waves $MM^{\prime }$ excited by each feeding element along the line in Figure 1 have the same phase, referred to as an in-phase wavefront. The direction of the beam maximum pattern is perpendicular to the in-phase wavefront [34].

In summary, when each feeding element remains in an ideal condition, the overall pattern model excited by the linear phased array feed can be expressed as follows:

(4) $$F\left(\theta ,\varphi \right)=\sum_{n=0}^{N-1}F_{e}\left(\psi_{n}\right)A_{n}e^{jn(\Delta \phi_{n}+\varphi_{n})}=\sum_{n=0}^{N-1}F_{e}\left(\psi_{n}\right)A_{n}e^{j\frac{2\pi }{\lambda }nd\left(\sin\;\theta \sin\;\varphi -\sin\;\theta_{B}\sin\;\varphi_{B}\right)}$$

### 2.2. Parabolic Cylindrical Reflector Pattern Model

**Figure 2:**
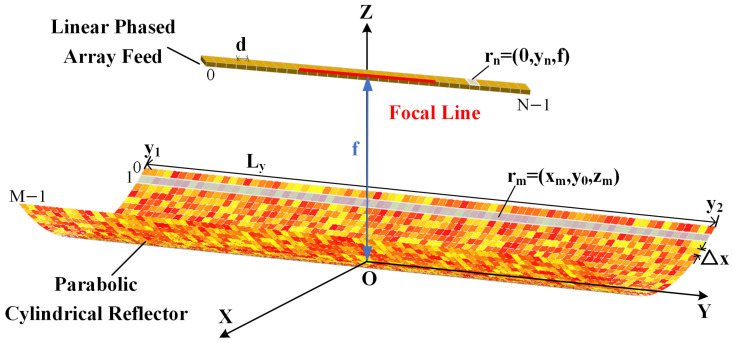
The parabolic cylindrical antenna system structure.

The parabolic cylindrical reflector is formed by translating a parabola along the normal of its plane. There are usually two types of feed: one places a point source at a specific location on the focal line; the other, employed in this study, is the phase center of the linear phased array feed, which is located on the focal line of the parabolic cylindrical reflector. In this case, the phase of the antenna aperture field remains in phase, and the overall structure is shown in Figure 2. To precisely describe the geometric relationship between the feed and the reflector, the vertex of the parabolic reflector is defined as the origin O(0,0,0) of the global coordinate system. The *Y* axis is parallel to the extension of the parabolic reflector’s focal line. The *Z* axis is perpendicular to the tangent plane at the reflector vertex and points toward the feed. The *X* axis is perpendicular to the *Y*-*Z* plane.

Perpendicular to the focal line, the parabolic cylindrical antenna system forms a fixed normal beam (without array scanning capability) due to significant reflector curvature variation and no feed distribution. Its radiation characteristics are jointly determined by the feed’s radiation performance and the antenna’s focusing properties [35]. Parallel to the focal line, the system has beamforming capability, with beam scanning achieved by adjusting the phase of the feeding elements to meet the requirements of various SAR imaging modes.

This study aims to construct a model capable of evaluating the impact of multi-source structural errors on far-field radiation patterns. To this end, the following assumptions are made based on high-frequency approximation theory. The validity of these assumptions is verified via FEKO simulations at the end of this section.

1. Mutual coupling between elements and multiple reflections are neglected: For the electrically large antennas investigated in this work, the PO method accurately characterizes the radiation of the main lobe and sidelobes. The pattern distortion caused by multi-source structural errors significantly outweighs perturbations from mutual coupling, making this assumption valid, particularly for high-frequency applications.

2. Scattering effects from reflector edges, transition zones, and shadow regions are neglected: In parabolic cylindrical antennas, energy is highly concentrated within the main beam. Consequently, the influence of edge diffraction fields on the main lobe and near sidelobes is minimal, rendering this approximation sufficient for most practical scenarios.

3. Feed blockage effects on the reflection path are ignored: This simplification allows the model to be directly adapted to offset-fed structures, which are widely used in SAR systems. Additionally, it serves as an ideal performance benchmark for center-fed structures (as shown in the figures in this paper) by excluding blockage effects. However, for scenarios involving severe feed blockage or requiring high-precision cross-polarization analysis, these factors should be addressed in future studies.

**Figure 3:**
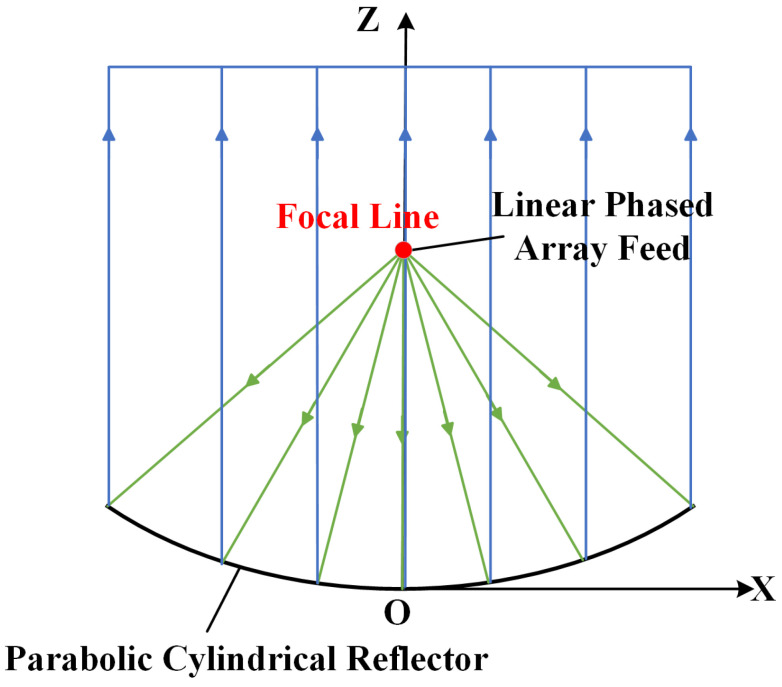
The reflection principle of a parabolic cylinder perpendicular to the focal line.

The reflection principle of a parabolic cylindrical reflector is shown in Figure 3. The linear phased array feed generates a sector wave centered on the focal line. After reflection by the parabolic cylindrical reflector, one-dimensional focusing is achieved in the direction perpendicular to the focal line. The reflected wavefront approximates a plane wave. It maintains its fan beam characteristic along the focal line and serves solely as a reflector without a focusing effect. The parabolic cylindrical antenna is generally a fan beam antenna and is suitable for a radar system with wavelengths ranging from centimeters to meters [17,35,36].

The specific processes of constructing the parabolic cylindrical antenna’s far-field pattern model based on the PO method are: First, calculate the equivalent surface current induced by the primary feed’s radiation field on the reflector. Second, compute the far-field radiation of the equivalent surface current using the radiation integral. Finally, derive the antenna’s far-field pattern.

Assuming the focal line is along the *Y* axis, the geometry of the parabolic cylindrical reflector is approximated as follows:

(5) $$z=\frac{x^{2}}{4f}$$

where *f* is the focal length. The element normal vector ${\hat{n}}_{m}$ at any point $P_{m}$ on the parabolic cylinder is represented by the gradient direction.

The incident wave electric field at a point on the parabolic cylindrical reflector for the *n*-th feeding element in a linear phased array feed system is as follows:

(6) $${\vec{E}}_{i,n}={\left(\frac{\left({\vec{r}}_{m}-{\vec{r}}_{n}\right)\;\cdot \;{\hat{u}}_{n}}{|{\vec{r}}_{m}-{\vec{r}}_{n}|}\right)}^{q}\;A_{n}e^{jn\Delta \phi_{B}}e^{-jk\left|{\vec{r}}_{m}-{\vec{r}}_{n}\right|}\frac{1}{\left|{\vec{r}}_{m}-{\vec{r}}_{n}\right|}{\hat{e}}_{p}$$

where $A_{n}$ is the excitation amplitude of the feeding element, Δ$\phi_{B}$ is the excitation phase of two adjacent feeding elements, ${\vec{r}}_{n}=\left(x_{n},y_{n},z_{n}\right)$ is the position vector of the *n*-th feeding element, ${\vec{r}}_{m}=\left(x_{m},y_{m},z_{m}\right)$ is the position vector of the *m*-th discrete element on the reflector, $\left|{\vec{r}}_{m}-{\vec{r}}_{n}\right|$ is the actual distance between the feeding element and the reflective surface element, ${\hat{u}}_{n}$ is the main radiation direction range vector of the feeding element, ${\hat{e}}_{p}$ is the element polarization vector describing the direction of the electric field vector in space. For a single polarized array, if the electric field is a horizontally polarized wave along the *X* axis, then ${\hat{e}}_{p}={\hat{e}}_{x}=\left(1,0,0\right)$; if the electric field is a vertically polarized wave along the *Z* axis, then ${\hat{e}}_{p}={\hat{e}}_{z}=\left(0,0,1\right)$.

According to the principle of field strength superposition, the total incident electric field at a discrete element on the parabolic cylindrical reflector, generated by the feeding array mechanism, is the vector superposition of the *N* electric fields of the elements, which can be described as follows.

(7) $$\begin{array}{c} {\vec{E}}_{i}=\sum_{n=0}^{N-1}{\vec{E}}_{i,n}=\sum_{n=0}^{N-1}{\left(\frac{\left({\vec{r}}_{m}-{\vec{r}}_{n}\right)\;\cdot \;{\hat{u}}_{n}}{|{\vec{r}}_{m}-{\vec{r}}_{n}|}\right)}^{q}\;A_{n}e^{jn\Delta \phi_{B}}e^{-jk\left|{\vec{r}}_{m}-{\vec{r}}_{n}\right|}\frac{1}{\left|{\vec{r}}_{m}-{\vec{r}}_{n}\right|}{\hat{e}}_{p}\\ \;={\hat{e}}_{p}\sum_{n=0}^{N-1}{\left(\frac{\left({\vec{r}}_{m}-{\vec{r}}_{n}\right)\;\cdot \;{\hat{u}}_{n}}{|{\vec{r}}_{m}-{\vec{r}}_{n}|}\right)}^{q}\;A_{n}e^{-jknd\sin\;\theta_{B}\sin\;\varphi_{B}}e^{-jk\left|{\vec{r}}_{m}-{\vec{r}}_{n}\right|}\frac{1}{\left|{\vec{r}}_{m}-{\vec{r}}_{n}\right|} \end{array}$$

According to the PO method, the equivalent current density at this position on the parabolic cylindrical reflector is shown in (8).

(8) $$\begin{array}{c} {\vec{J}}_{s}\left({\vec{r}}_{m}\right)=2{\hat{n}}_{m}\times {\vec{H}}_{i}\left({\vec{r}}_{m}\right) \end{array}$$

where ${\hat{n}}_{m}$ is the element normal vector at point, ${\vec{H}}_{i}\left({\vec{r}}_{m}\right)$ is the incident magnetic field given by (9).

(9) $$\begin{array}{c} {\vec{H}}_{i}\left({\vec{r}}_{m}\right)=\frac{1}{\eta }{\hat{e}}_{i}\times {\vec{E}}_{i}\left({\vec{r}}_{m}\right) \end{array}$$

where $\eta =\sqrt{\frac{\mu }{\varepsilon }}$ is the free space wave impedance. ${\hat{e}}_{i}$ is the incident wave element vector. When the linear phased array feed is excited, it is typically represented by the element vector pointing from the feed center to the reflector, i.e., ${\hat{e}}_{i}\approx \frac{\vec{r}}{\left|\vec{r}\right|}$.

The far-field pattern is obtained by integrating the equivalent current over the reflector, as written in (10).

(10) $$\begin{array}{c} \vec{E}\left(\theta ,\varphi \right)\propto \underset{S}{\int \int }{\vec{J}}_{s}\left({\vec{r}}_{m}\right)e^{jk\left(\hat{R}\;\cdot \;{\vec{r}}_{m}\right)}dS \end{array}$$

where $\hat{R}=\left(\sin\;\theta \cos\;\varphi ,\sin\;\theta \sin\;\varphi ,\cos\;\theta \right)$ is the element vector of the far-field observation direction of the incident wave after reflection in the spherical coordinate system, and dS is the area element of the reflector.

Substituting the total incident field (7), the equivalent current (8) and (9) into the far-field integral expression (10), the far-field pattern yield is shown in (11):

(11) $$\begin{array}{c} \begin{array}{c} \vec{E}\left(\theta ,\varphi \right) \end{array}=\int \int_{S}\left[{\hat{n}}_{m_{xy}}\times \left({\hat{e}}_{i}\times {\hat{e}}_{p}\right)\right]\\ \;\cdot \;\left[\sum_{n=0}^{N-1}\begin{array}{c} {\left(\frac{\left({\vec{r}}_{m_{xy}}-{\vec{r}}_{n}\right)\;\cdot \;{\hat{u}}_{n}}{|{\vec{r}}_{m_{xy}}-{\vec{r}}_{n}|}\right)}^{q}\; \end{array}A_{n}e^{-jknd\sin\;\theta_{B}\sin\;\varphi_{B}}e^{-jk\left|{\vec{r}}_{m_{xy}}-{\vec{r}}_{n}\right|}\frac{1}{\left|{\vec{r}}_{m_{xy}}-{\vec{r}}_{n}\right|}\right]e^{jk\left(\hat{R}\cdot {\vec{r}}_{m_{xy}}\right)}dS\\ \;=\sum_{m_{x}=0}^{M_{x}-1}\sum_{m_{y}=0}^{M_{y}-1}\sum_{n=0}^{N-1}A_{m_{xy}n}e^{j\phi_{m_{xy}n}} \end{array}$$

where $\left[{\hat{n}}_{m_{xy}}\times \left({\hat{e}}_{i}\times {\hat{e}}_{p}\right)\right]$ is the polarization vector factor containing the normal vector of the reflector, the incident direction, and the polarization direction; $A_{m_{xy}n}$ is the overall amplitude factor of the model; $\phi_{m_{xy}n}$ is the overall phase factor of the model consisting of four components: the scanning phase $\phi_{scan}$ of the feed, the actual path propagation phase $\phi_{path}$ from the feed to the reflector, the actual projection phase $\phi_{proj}$ from the reflector to the far-field, and the polarization vector phase $\phi_{pol}$.

In order to more accurately discuss the impact of structural errors on the electromagnetic characteristics of the reflected wave, the parabolic cylindrical reflector is discretized in one-dimension along the *X* axis with the largest curvature change. It is divided into *M* grid cells, with the cell center at $x_{m}$ and the cell discretization length as $\Delta x_{m}$. Continuous integration is performed along the focal line direction where the curvature change is approximately constant, with an integration length of $L_{y}$. It can be further obtained as follows:

(12) $$\left\{\begin{array}{c} F_{r}\left(\theta ,\varphi \right)\begin{array}{c} \approx \end{array}\sum_{m=0}^{M-1}\left[\int_{y_{1}}^{y_{2}}\sum_{n=0}^{N-1}\left[A_{mn}e^{j\phi_{mn}\left(y\right)}\right]dy\right]\\ A_{mn}=A_{n}\;\cdot \;\left|\left[{\hat{n}}_{m}\times \left({\hat{e}}_{i}\times {\hat{e}}_{p}\right)\right]\right|\;\cdot \;\Delta x_{m}\begin{array}{c} {\left(\frac{\left({\vec{r}}_{m}-{\vec{r}}_{n}\right)\;\cdot \;{\hat{u}}_{n}}{|{\vec{r}}_{m}-{\vec{r}}_{n}|}\right)}^{q}\frac{1}{\left|{\vec{r}}_{m}-{\vec{r}}_{n}\right|} \end{array}\\ \phi_{mn}\left(y\right)=\phi_{scan}+\phi_{path}+\phi_{proj}+\phi_{pol}\\ =-knd\;\sin\;\theta_{B}\sin\;\varphi_{B}-k\left|{\vec{r}}_{m}\left(y\right)-{\vec{r}}_{n}\right|+k\left(\hat{R}\;\cdot \;{\vec{r}}_{m}\left(y\right)\right)+∠\left[{\hat{n}}_{m}\times \left({\hat{e}}_{i}\times {\hat{e}}_{p}\right)\right] \end{array}\right.$$

### 2.3. Comprehensive Analysis and Modeling of Errors in Linear Array-Fed Parabolic Cylindrical Antennas

This research focuses on the installation position error which is a type of structural error, defined as the random spatial offset of feeding elements relative to their designed positions during installation or application. This error directly induces non-ideal spatial phase differences in far-field radiation by changing the geometric paths between array elements [37,38], without changing the in-array phase differences controlled by the phase shifter. The adverse effect of the feed installation position error on the antenna pattern is significant, typically including main lobe offset, gain reduction, and sidelobe level rise [39].

**Figure 4:**
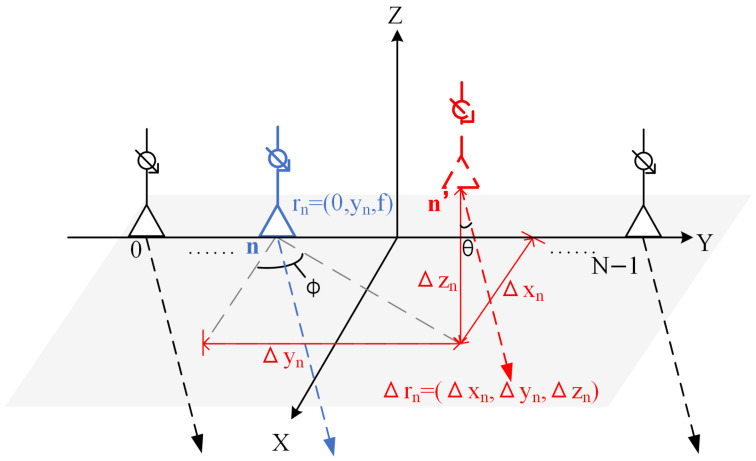
The installation position error of a certain feeding element in a linear phased array.

Under ideal condition, assuming the linear feed array is positioned at global coordinate $\left(0,y_{n},f\right)$, with the primary radiation direction along the −Z axis pointing toward the reflector, a certain feed unit generates an error offset $\Delta {\vec{r}}_{n}=\left(\Delta x_{n},\Delta y_{n},\Delta z_{n}\right)$ as shown in Figure 4.

For each feeding element with installation-position errors, an additional path difference is introduced by its position deviation. With these errors taken into account, the actual position of the *n*-th feeding element is as follows:

(13) $${{\vec{r}}_{n}}^{\prime }={\vec{r}}_{n}+\Delta {\vec{r}}_{n}=\left(\Delta x_{n},y_{n}+\Delta y_{n},f+\Delta z_{n}\right)$$

Due to the change in the spatial position between the feed, the spatial phase difference varies. Under the influence of errors, the overall pattern model of the linear phased array feed becomes the following:

(14) $$\begin{array}{c} F^{\prime }\left(\theta ,\varphi \right)=\sum_{n=0}^{N-1}\begin{array}{c} {F_{e}}^{\prime }\left({\psi_{n}}^{\prime }\right) \end{array}A_{n}e^{j({\Delta \phi_{n}}^{\prime }+n\Delta \phi_{B})}\\ \;=\sum_{n=0}^{N-1}\begin{array}{c} {F_{e}}^{\prime }\left({\psi_{n}}^{\prime }\right) \end{array}A_{n}e^{j\frac{2\pi }{\lambda }\left[\Delta x_{n}\sin\;\theta \cos\;\varphi +\left(nd+\Delta y_{n}\right)\sin\;\theta \sin\;\varphi +\Delta z_{n}\cos\;\theta -nd\sin\;\theta_{B}\sin\;\varphi_{B}\right]} \end{array}$$

The surface error of the parabolic cylindrical reflector is another important factor affecting antenna performance. Its causes are complex and may involve multiple factors, such as self-weight, manufacturing processes, and environmental thermal effects [40,41]. The normal structural error, the focus of this research, is defined as the deviation between the local normal direction of the reflector and its ideal designed value. This error disrupts the incident path, inducing phase distortion in the reflected wavefront. Consequently, it leads to several performance degradations: reduced antenna gain, beam pointing deviation, sidelobe elevation, and beam broadening [42].

A partitioned superposition approach is proposed to solve this problem. First, the reflector is discretized one-dimensionally along the direction with dramatic curvature changes. Next, the impact of the normal structural error of each discrete element on the local radiation field is evaluated. Finally, coherent superimposition of the radiation fields from all discrete elements yields a pattern model incorporating the reflector’s overall normal structural error [42,43].

**Figure 5:**
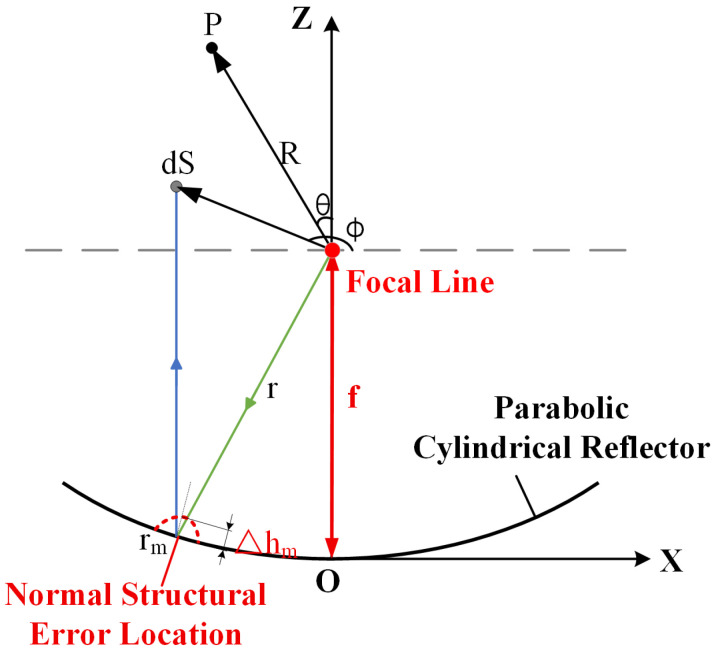
Structural errors at a certain grid on the parabolic cylindrical reflector.

Assuming that structural errors in the reflector cause a discrete element to deviate from its theoretical position, resulting in a small normal offset $\Delta h_{m}$, where positive direction indicates a protrusion, and the negative direction indicates a depression, as shown in Figure 5.

When the center position of the ideal grid is slightly offset due to local normal errors, the actual grid center position $\begin{array}{c} {\vec{r}}_{m}^{\prime } \end{array}=\left(x_{m}^{\prime },y_{m}^{\prime },z_{m}^{\prime }\right)$ is as follows:

(15) $$\begin{array}{c} {\vec{r}}_{m}^{\prime } \end{array}=\begin{array}{c} {\vec{r}}_{m} \end{array}+\;\Delta h_{m}{\hat{n}}_{m}$$

Reflector normal structural error induces dual-phase perturbation in the far-field pattern: it not only modifies the actual incident wave path length from the feed to the error location, but also varies the reflected path from this location to the far-field. The resulting phase distortion arises from the coherent superposition of these two phase perturbations.

**Figure 6:**
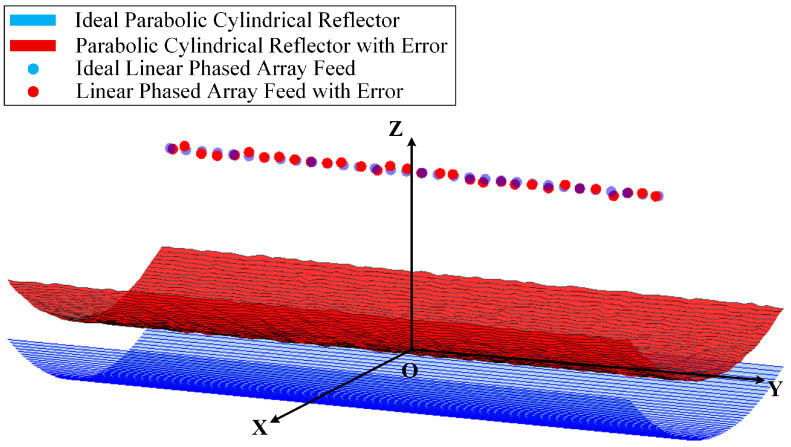
A parabolic cylindrical antenna system comparing ideal and error.

A simulation model of the linear array-fed parabolic cylindrical antenna system is established by incorporating the three-dimensional random position deviation of the phased array feed and random normal deformation of the reflector (Figure 6). The blue model represents the ideal structure without errors, and the red model corresponds to the structure with the two aforementioned errors. A slight *Z*-axis translation of the ideal reflector is applied for visual clarity only, with no impact on simulation physics or results.

The coexistence of multi-source structural errors exerts a dual modulation effect on the antenna radiation field. The path vector from the feed to the reflector is as follows:

(16) $$\begin{array}{c} {\vec{R}}_{mn}^{\prime }={\vec{r}}_{m}^{\prime }-{\vec{r}}_{n}^{\prime }=\left(x_{m}^{\prime }-\Delta x_{n},\;y_{m}^{\prime }-\left(y_{n}+\Delta y_{n}\right),\;z_{m}^{\prime }-\left(f+\Delta z_{n}\right)\right) \end{array}$$

On the one hand, the feed’s position error first modifies the initial phase of the incident wave across the reflector. Interacting with the reflector’s normal errors, it induces secondary perturbations in the reflected wave’s path and phase, which collectively disrupt the reflected wave’s coherent superposition and thus alter the far-field pattern’s radiation characteristics. In the parabolic cylindrical pattern model, it is reflected that the precise path phase $\phi_{path}$ from the feed to the reflector and the far-field projection phase $\phi_{proj}$ change respectively, which lead to the two key phase factors in (12), respectively becoming $e^{-jk\left|{\vec{r}}_{m}^{\prime }-{\vec{r}}_{n}^{\prime }\right|}$ and $e^{jk\left(\hat{R}\cdot {\vec{r}}_{m}^{\prime }\right)}$, produce nonlinear phase distortion.

Moreover, structural errors alter the relative position between the feed and the reflector. This alters the incident field amplitude by varying the local illumination angle and propagation path length. Consequently, the radiation pattern function of the feeding element is expressed as follows:

(17) $$\begin{array}{c} F_{e}^{\prime }\left(\psi_{mn}^{\prime }\right)=\cos^{q}\left(\psi_{mn}^{\prime }\right)=\frac{{\left({\vec{R}}_{mn}^{\prime }\;\cdot \;{\hat{u}}_{n}^{\prime }\right)}^{q}}{{\left|{\vec{R}}_{mn}^{\prime }\right|}^{q}}=\frac{{\left(z_{n}^{\prime }-z_{m}^{\prime }\right)}^{q}}{{\left|{\vec{r}}_{m}^{\prime }-{\vec{r}}_{n}^{\prime }\right|}^{q}} \end{array}$$

The direct integration pattern model of the parabolic cylindrical antenna is as shown in (18):

(18) $$\left\{\begin{array}{c} F_{r}^{\prime }\left(\theta ,\varphi \right)=\sum_{m=0}^{M-1}\left[\int_{y_{1}}^{y_{2}}\sum_{n=0}^{N-1}\left[A_{mn}^{\prime }e^{j\phi_{mn}^{\prime }\left(y\right)}\right]dy\right]\\ A_{mn}^{\prime }=A_{n}\;\cdot \;\left|\left[{\hat{n}}_{m}\times \left({\hat{e}}_{i}\times {\hat{e}}_{p}\right)\right]\right|\;\cdot \;\Delta x_{m}\begin{array}{c} \frac{{\left(z_{n}^{\prime }-z_{m}^{\prime }\right)}^{q}}{{\left|{\vec{r}}_{m}^{\prime }-{\vec{r}}_{n}^{\prime }\right|}^{q+1}} \end{array}\\ \phi_{mn}^{\prime }\left(y\right)=-knd\;\sin\;\theta_{B}\sin\;\varphi_{B}-k\left|{\vec{r}}_{m}^{\prime }\left(y\right)-{\vec{r}}_{n}^{\prime }\right|+k\left(\hat{R}\;\cdot \;{\vec{r}}_{m}^{\prime }\left(y\right)\right)+∠\left[{\hat{n}}_{m}\times \left({\hat{e}}_{i}\times {\hat{e}}_{p}\right)\right] \end{array}\right.$$

### 2.4. Direct Integration Model Accuracy Validation

**Figure 7:**
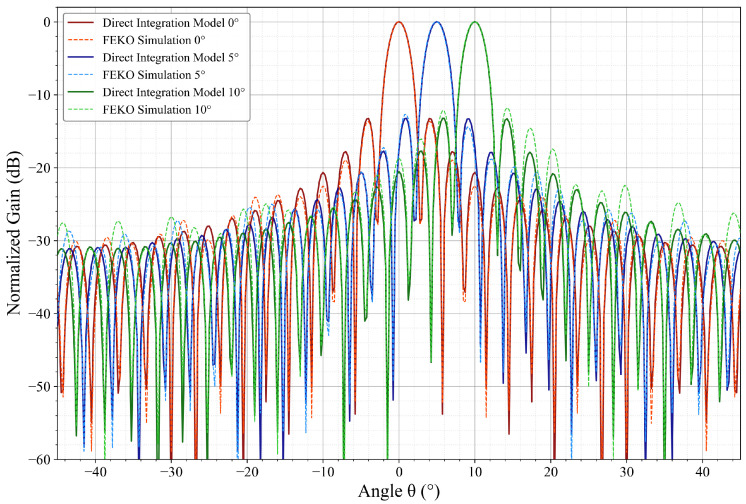
Comparison of the direct integration model and the FEKO simulation pattern.

To validate the accuracy of the direct integration model and ensure that the assumptions made during its derivation yield acceptable computational precision, a parabolic cylindrical antenna model was established using FEKO 2021.2. The radiation patterns calculated via the MLFMM were utilized as the reference benchmark. Specific beam scanning angles $\theta_{scan}=$ 0°, 5°, 10° were selected for evaluation. Figure 7 presents a comparison of the normalized radiation patterns between the direct integration model and the FEKO simulation under ideal conditions.

It can be observed from Figure 7 that the direct integration model achieves a high degree of agreement within the main lobe. Furthermore, the trends in the first sidelobe levels and null positions remain consistent with the reference. The deviations introduced by the model assumptions are primarily concentrated in the far sidelobe regions. Therefore, they do not affect the investigation of electrical performance in critical regions. To quantitatively evaluate the precision of the model, several key metrics were selected for calculation, and the results are presented in Table 1.

Quantitative analysis indicates that within the typical scanning range of 0° to 10°, the variation in beamwidth remains within 0.1° (approximately 3%). The Root Mean Square Error (RMSE) of the main lobe between the two methods is less than 0.2 dB across all scanning angles. Furthermore, the beam pointing deviation remains zero throughout the test range. These results demonstrate that the proposed direct integration model accurately reproduces the antenna radiation characteristics, thereby satisfying the requirements for subsequent derivations. However, at large scanning angles, the accuracy of the direct integration model decreases slightly. This is attributed to the exclusion of mutual coupling, diffraction, and blockage effects in the model assumptions. Consequently, further research is necessary to refine the model for broader adaptability across various scanning modes.

## 3. Simplified Pattern Modeling Based on the Fresnel Approximation Principle

After comprehensive error analysis, the direct integration model shows complex multiple integrals and multi-parameter coupling. Direct numerical integration for its solution leads to high computational costs and hinders subsequent applications and model optimization. To convert the computational complexity from numerical integration to analytical function evaluation, Equation (18) is simplified, with the specific approaches outlined below:

1. In contrast to the rapid oscillation of the phase factor along the focal line integration path, the amplitude variations induced by the element radiation pattern and the spatial path loss factor in the direct integration model are relatively slow. To transform the direct integration model into the standard form of Fresnel integrals, the slowly varying amplitude term is approximated as a constant.

2. The structural errors of the reflector are typically much smaller than the focal length, wavelength, and observation distance. Therefore, the path length variations introduced by these errors primarily manifest as phase distortions. For small-scale deformations, the perturbations to the amplitude and polarization vectors caused by structural errors are considered negligible.

3. Since the region of interest is concentrated on the main lobe and near sidelobes, the paraxial condition is satisfied. Accordingly, the Fresnel approximation is employed to compute the continuous integration along the focal line. This approach simplifies the calculation process while maintaining high computational accuracy.

### 3.1. Fresnel Approximation Principle

Derived from the expansion and simplification of the Huygens–Fresnel diffraction integral, the Fresnel approximation is a widely used method in optics and in the analysis of electromagnetic wave propagation. For a wave propagating in a uniform medium under the paraxial approximation condition, the distance *R* between the observation point and the wave source is expanded into a Taylor series approximation form:

(19) $$\begin{array}{c} R=\sqrt{x^{2}+y^{2}+z^{2}}=z\left[1+\frac{x^{2}+y^{2}}{2z^{2}}+\frac{{\left(x^{2}+y^{2}\right)}^{2}}{8z^{4}}+\ldots \right] \end{array}$$

For the phase factor, when the wave’s dominant propagation distance *z* is sufficiently large, the expansion of (19) can be truncated to the first two terms and ignore the higher terms. With a typical approximation accuracy of 99% and no significant phase errors introduced, this method exhibits low computational complexity and modest data volume requirements, which defines the Fresnel approximation principle [44,45].

The diffraction field expression obtained via the Fresnel approximation principle has an integrand kernel consisting of sine and cosine functions with a quadratic phase term. Its integral result can be directly denoted using the Fresnel integral functions S(x) and C(x). The definition of the Fresnel integral functions is as follows:

(20) $$\begin{array}{cc} S(x) & =\int_{0}^{x}\sin\;\left(\frac{\pi }{2}t^{2}\right)dt\\ C(x) & =\int_{0}^{x}\cos\;\left(\frac{\pi }{2}t^{2}\right)dt \end{array}$$

The Fresnel integral functions are the standard forms presented by specific integral expressions after applying the Fresnel approximation. They are special functions shared by multiple disciplines, such as PO, microwave technology, and antennas [46]. After applying the Fresnel approximation, the integral can be cast into the standard Fresnel integral form. Its value either computable via analytical approximation functions or directly accessible from standard Fresnel function library, yielding a notable improvement in numerical computation and analysis efficiency.

### 3.2. Fresnel Simplified Pattern Modeling

For electrically large objects such as the parabolic cylindrical antennas (λ≪l), dielectric properties and geometric parameters typically vary slowly [23]. The Fresnel theory and direct integration model are both mathematically based on electromagnetic-wave superposition integrals and share many similarities. Based on the fundamental principles of Fresnel integration, the amplitude term in the direct integration model varies slowly relative to the phase term across the integration interval. It is treated as a constant and factored out of the integral. For the rapidly varying phase term, the Fresnel approximation is applied by performing a Taylor series expansion on the phase factor with respect to the integration variable. This approximates the complex phase as a quadratic function, transforming the original expression into a standard Fresnel integral form, which significantly reduces computational complexity. This method retains the dominant quadratic phase term, which is consistent with electromagnetic diffraction and propagation laws. Additionally, the method preserves the equivalent current integral structure, enabling efficient model approximation.

In the core phase term $\left|{\vec{r}}_{m}^{\prime }\left(y\right)-{\vec{r}}_{n}^{\prime }\right|$ affected by the comprehensive errors in (18), identifying parameters related to the integral direction *y* and expressing them as follows:

(21) $$\begin{array}{c} \left|{{\vec{r}}^{\prime }}_{m}\left(y\right)-{{\vec{r}}^{\prime }}_{n}\right|=\sqrt{{\left({x^{\prime }}_{m}-\Delta x_{n}\right)}^{2}+{\left(y-\left(nd+\Delta y_{n}\right)\right)}^{2}+{\left({z^{\prime }}_{m}-\Delta z_{n}\right)}^{2}}\\ \;=\sqrt{D_{mn}^{2}+{\left(y-{y^{\prime }}_{n}\right)}^{2}}=D_{mn}\sqrt{1+\frac{{\left(y-{y^{\prime }}_{n}\right)}^{2}}{D_{mn}^{2}}} \end{array}$$

where ${D_{mn}}^{2}={\left(x_{m}^{\prime }-\Delta x_{n}\right)}^{2}+{\left(z_{m}^{\prime }-\Delta z_{n}\right)}^{2}$ is a parameter independent of the integral direction.

Using the Fresnel approximation method, Equation (21) is approximately expanded in the form of a Taylor series, ignoring the quadratic parts of the integral variable [44], we obtain (22).

(22) $$\left|{\vec{r}}_{m}^{\prime }\left(y\right)-{\vec{r}}_{n}^{\prime }\right|\approx D_{mn}\left(1+\frac{{\left(y-y_{n}^{\prime }\right)}^{2}}{2{D_{mn}}^{2}}\right)=D_{mn}+\frac{{\left(y-y_{n}^{\prime }\right)}^{2}}{2D_{mn}}$$

Substitute the parameter (22) after the Fresnel approximation into the overall phase factor in (18), and denote the integral part as $\phi \left(y\right)$ in (23).

(23) $$\begin{array}{c} \phi \left(y\right)=-k\left|{\vec{r}}_{m}^{\prime }\left(y\right)-{\vec{r}}_{n}^{\prime }\right|+k\left(\hat{R}\;\cdot \;{\vec{r}}_{m}^{\prime }\left(y\right)\right)\\ \;\approx -k\left[D_{mn}+\frac{y^{2}-2y{y^{\prime }}_{n}+{\left({y^{\prime }}_{n}\right)}^{2}}{2D_{mn}}\right]+k\left({\hat{R}}_{x}{x^{\prime }}_{m}+{\hat{R}}_{y}y+{\hat{R}}_{z}{z^{\prime }}_{m}\right) \end{array}$$

Further, the parameter terms highly correlated with the integral direction are expressed as $\phi_{y}\left(y\right)$ in (24).

(24) $$\phi_{y}\left(y\right)=-k\left[\frac{y^{2}-2yy_{n}^{\prime }}{2D_{mn}}\right]+k{\hat{R}}_{y}y=-\frac{k}{2D_{mn}}y^{2}+\left(\frac{ky_{n}^{\prime }}{D_{mn}}+k{\hat{R}}_{y}\right)y=\alpha_{mn}y^{2}+\beta_{mn}y$$

where $\alpha_{mn}=-\frac{k}{2D_{mn}}$, $\beta_{mn}=\left(\frac{ky_{n}^{\prime }}{D_{mn}}+k{\hat{R}}_{y}\right)$.

The Fresnel approximation function integrated continuously along the *Y*-axis direction can be expressed as $F_{y,mn}$ in (25).

(25) $$F_{y,mn}=\int_{y_{1}}^{y_{2}}e^{j\left(\alpha_{mn}y^{2}+\beta_{mn}y\right)}dy$$

By formulating the quadratic polynomial of the exponential term in (25), we can obtain the following:

(26) $$F_{y,mn}=e^{-j\left(\frac{\beta_{mn}^{2}}{4\alpha_{mn}}\right)}\int_{y_{1}}^{y_{2}}e^{j\alpha_{mn}{\left[y+\left(\frac{\beta_{mn}}{2\alpha_{mn}}\right)\right]}^{2}}dy$$

To transform the integral kernel into the standard form of $e^{j\frac{\pi }{2}t^{2}}$, a new integral variable *t* is introduced as follows:

(27) $$t=\sqrt{\frac{2\alpha_{mn}}{\pi }}\;\cdot \;\left(y+\frac{\beta_{mn}}{2\alpha_{mn}}\right)$$

By using the new integral variable to achieve variable substitution and substituting the result into Equation (26), we can obtain the following:

(28) $$\begin{array}{cc} F_{y,mn} & =\sqrt{\frac{\pi }{2\alpha_{mn}}}\;\cdot \;e^{-j\left(\frac{\beta_{mn}^{2}}{4\alpha_{mn}}\right)}\;\cdot \;\int_{t_{1}}^{t_{2}}e^{j\frac{\pi }{2}t^{2}}dt\\ t_{1} & =\sqrt{\frac{2\alpha_{mn}}{\pi }}\;\cdot \;\left(y_{1}+\frac{\beta_{mn}}{2\alpha_{mn}}\right)\\ t_{2} & =\sqrt{\frac{2\alpha_{mn}}{\pi }}\;\cdot \;\left(y_{2}+\frac{\beta_{mn}}{2\alpha_{mn}}\right) \end{array}$$

Based on Euler’s formula, the above integral can be decomposed into the standard Fresnel integral forms shown in (20):

(29) $$\int_{t_{1}}^{t_{2}}e^{j\frac{\pi }{2}t^{2}}dt=\left[C\left(t_{2}\right)-C\left(t_{1}\right)\right]+j\left[S\left(t_{2}\right)-S\left(t_{1}\right)\right]$$

Through the above matching method and variable substitution, the Fresnel approximation form (25) is successfully transformed into the standard Fresnel integral forms, achieving the purpose of evaluating using analytical functions and greatly improving the computational efficiency of the model.

The other phase term independent of the integral direction can be comprehensively expressed as ${\phi^{\prime \prime }}_{mn}$ in (30).

(30) $${\phi^{\prime \prime }}_{mn}=-knd\;\sin\;\theta_{B}\sin\;\varphi_{B}-kD_{mn}-k\left(\frac{{\left(y_{n}^{\prime }\right)}^{2}}{2D_{mn}}\right)+k\left({\hat{R}}_{x}x_{m}^{\prime }+{\hat{R}}_{z}z_{m}^{\prime }\right)$$

Combining Equations (18) and (28)–(30), the simplified pattern model after Fresnel approximation can be obtained as shown in (31).

(31) $$\begin{array}{cc} F_{r}^{\prime }\left(\theta ,\varphi \right) & \approx \sum_{m=0}^{M-1}\sum_{n=0}^{N-1}\left[A_{mn}e^{j\phi_{mn}^{\prime \prime }}F_{y,mn}\right]\\ & =\sum_{m=0}^{M-1}\sum_{n=0}^{N-1}[\begin{array}{c} A \end{array}\;\cdot \;e^{jk(-nd\sin\;\theta_{B}\sin\;\varphi_{B}-D_{mn}-\frac{{\left(y_{n}^{\prime }\right)}^{2}}{2D_{mn}}+\left({\hat{R}}_{x}x_{m}^{\prime }+{\hat{R}}_{z}z_{m}^{\prime }\right))}\\ & \;\cdot \;\sqrt{\frac{\pi }{2\alpha_{mn}}}\;\cdot \;e^{-j\left(\frac{\beta_{mn}^{2}}{4\alpha_{mn}}\right)}\;\cdot \;\left[\left(C\left(t_{2}\right)-C\left(t_{1}\right)\right)+j\left(S\left(t_{2}\right)-S\left(t_{1}\right)\right)\right]] \end{array}$$

### 3.3. Simplified Model Checking and Compensation Analysis

#### 3.3.1. Simulation Parameters Setting

The parabolic cylindrical antenna’s specific physical parameters for model accuracy verifications and simulations are listed in Table 2.

To mitigate the contingency associated with single random error samples and ensure the scientific reliability of the impact assessments, the Monte Carlo method is employed for simulation analyses. By performing repeated numerical simulations on large samples of randomly generated errors, this approach retrieves the statistical characteristics of the antenna pattern across all scanning angles. It serves as a classic and effective tool for evaluating the impact of random factors on complex systems [43,47]. Especially for nonlinear comprehensive error effects on antenna performance, the Monte Carlo method enables the direct simulation of statistical distributions and error transfer effects. This ensures both the convergence and reliability of the simulation results.

Three error levels (low, medium, and high) are defined to comprehensively cover structural error perturbations ranging from minor to significant. This configuration provides sufficient data support for the subsequent Monte Carlo simulations. The feed error primarily originates from installation position deviations of discrete elements, which are characterized by a small spatial variation scale. Conversely, the reflector error features spatially continuous correlation and is susceptible to factors such as deployment deformation and environmental conditions during installation. Given similar manufacturing tolerances, the actual physical scale of reflector errors is typically larger than that of feed errors. To reflect the physical characteristics of these two error types, distinct scaling baselines are adopted for each level. Table 3 presents the Root Mean Square (RMS) values for the random structural errors of the feed and reflector. All error values are normalized by wavelength to enhance comparability and generalizability of the results.

#### 3.3.2. Simplified Model Accuracy Verification

When employing the Fresnel approximation to simplify the antenna pattern, neglecting phase terms higher than the quadratic term may impact model accuracy. This truncation results in a discrepancy between the Fresnel-simplified pattern and the direct-integration pattern. The primary phase term ignored in this approximation is given by:

(32) $$\Delta \phi \left(y\right)=k\;\cdot \;\frac{{\left(y-y_{n}^{\prime }\right)}^{4}}{8D_{mn}^{3}}$$

Equation (32) shows that the Fresnel approximation-induced intrinsic deviation depends primarily on antenna physical parameters (e.g., wavelength, frequency, and aperture size).

To verify that the simplified model effectively enhances computational efficiency while satisfying accuracy requirements for practical applications, a comparative analysis was conducted. Specifically, the normalized amplitude and phase of the Fresnel Simplified Model were compared against those of the direct integration model under various error conditions.

**Figure 8:**
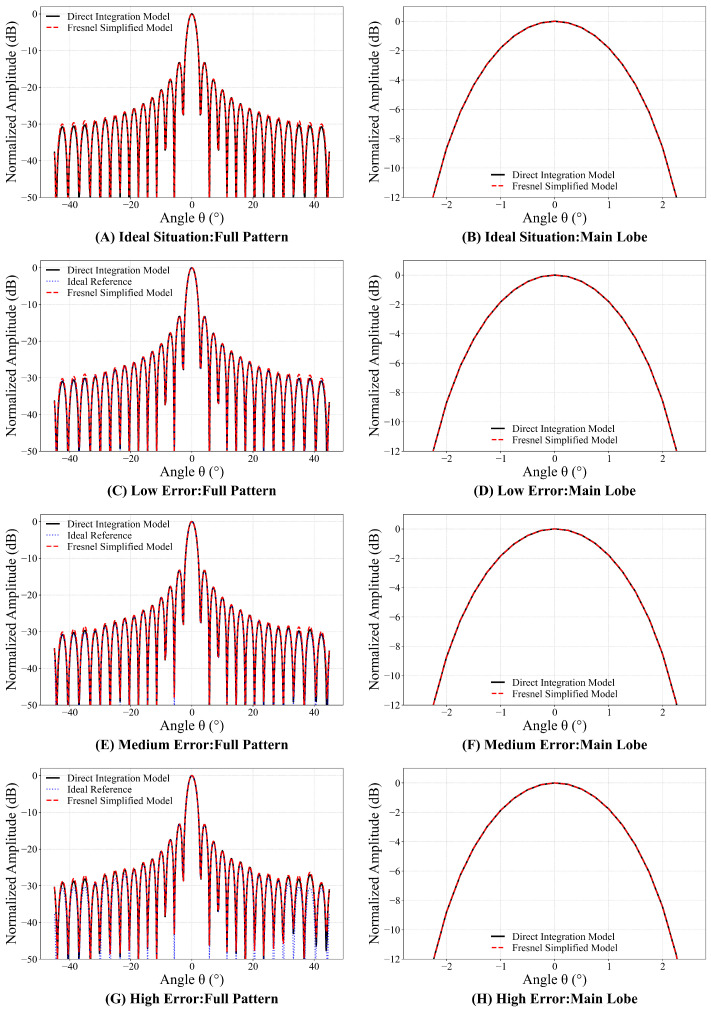
Comparison of the direct integration model and the Fresnel Simplified Model normalized patterns.

Figure 8 illustrates the comparison results of the normalized radiation patterns between the two models under these different error scenarios.

The results presented in Figure 8 indicate that the Fresnel Simplified Model effectively predicts the primary features of the radiation pattern. Despite the influence of multi-source structural errors, the simplified model’s overall prediction trend is highly consistent with that of the direct integration model. In particular, the shape and position of the main lobe and the first sidelobe demonstrate remarkable agreement. The discrepancies introduced by the Fresnel Simplified Model are primarily manifested as an elevation in far sidelobe levels and inconsistencies in null depths. These deviations have a negligible effect on the primary radiation performance of the antenna.

**Figure 9:**
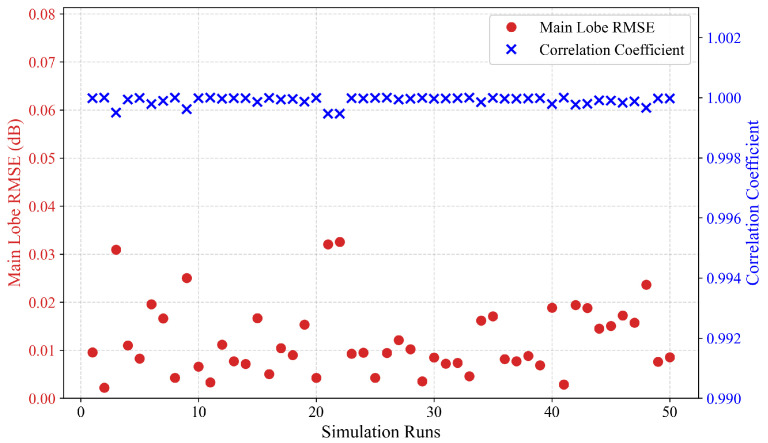
Statistical distribution of single run simulation accuracy for the model.

Since the average radiation pattern derived from Monte Carlo simulations may mask specific details of local beam distortion, a sample-by-sample fitting error statistical analysis was conducted on high error simulation samples to enhance the credibility of the model validation. The statistical results regarding the individual RMSE within the −3 dB main lobe width, and the waveform correlation coefficient between the Fresnel Simplified Model and the direct integration model are presented in Figure 9.

Across all random error samples, the simplified model exhibited exceptional computational stability. Specifically, the RMSE within the main lobe for individual samples remained consistently low, yielding an average RMSE of 0.0120 dB and a maximum of only 0.0325 dB. Moreover, the correlation coefficient for all samples consistently exceeded 0.9994. These results indicate that the simplified Fresnel model maintains high-fidelity predictions of antenna radiation characteristics, even under complex structural deformations, thereby satisfying computational accuracy requirements.

**Figure 10:**
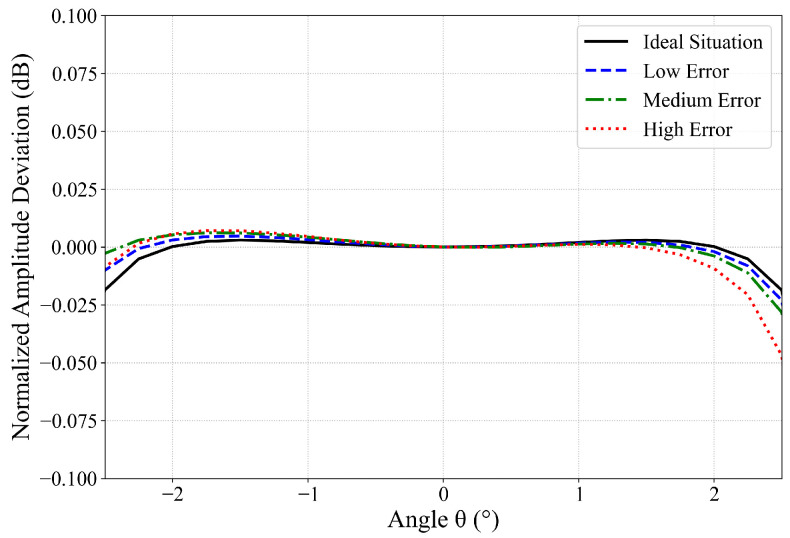
Normalized amplitude deviation results of the main lobe between the direct integration model and the Fresnel Simplified Model.

To visually demonstrate the high precision achieved by the simplified model, the normalized amplitude deviations between the Fresnel Simplified Model and the direct integration model within the main lobe range are illustrated in Figure 10.

The results indicate that within the core region of the main lobe, the amplitude deviation between the Fresnel Simplified Model and the direct integration model remains at an extremely low level across all error categories. Specifically, in the paraxial region, the deviation is negligible, demonstrating exceptional approximation accuracy. As the angle increases, the deviation rises slightly and exhibits a positive correlation with the magnitude of the structural errors. Within the −10 dB beamwidth, the normalized amplitude RMSE between the two models is less than 0.01 dB, with a maximum normalized amplitude deviation of less than 0.05 dB. A summary of selected statistical results is presented in Table 4.

**Figure 11:**
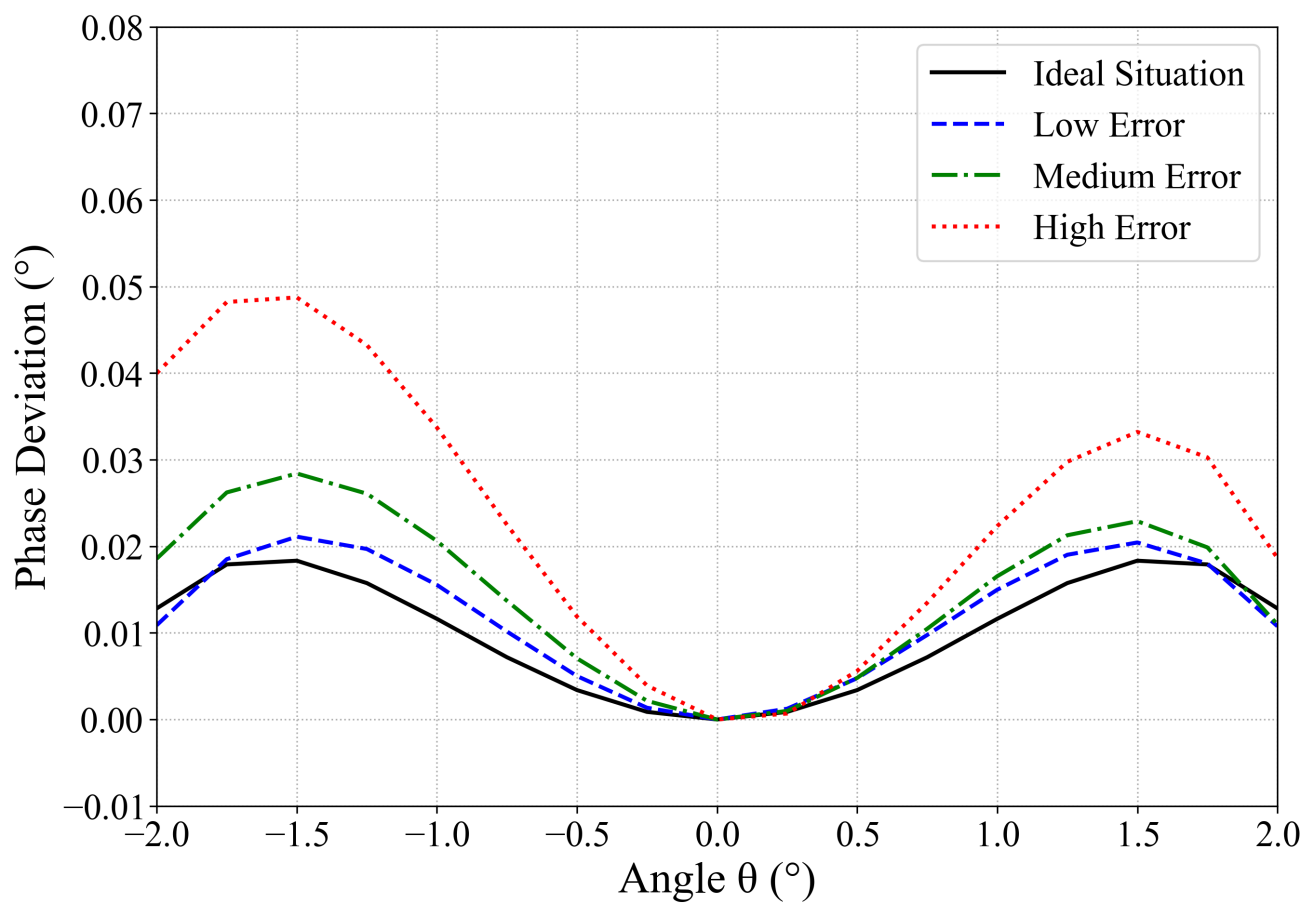
Phase deviation results of the main lobe between the direct integration model and the Fresnel Simplified Model.

To verify the phase accuracy of the simplified model on the main lobe, the phase deviation distribution results between the Fresnel Simplified Model and the direct integration model under different error conditions are shown in the Figure 11.

The results demonstrate that the Fresnel Simplified Model accurately reproduces the physical characteristics of the antenna pattern’s phase distribution within the main lobe region. The phase curves of both models exhibit consistent trends and similar variation patterns. As the error level increases, the phase deviation within the main lobe shows a slight increase. However, the overall magnitude of variation remains minimal. Under various error conditions, the RMSE of the main lobe phase is less than 0.03°, and the maximum phase deviation is less than 0.05°. These results confirm the extremely high phase accuracy of the model. Selected statistical results are summarized in Table 5.

Since the fundamental objective of the Fresnel approximation is to reduce the computational complexity of the direct integration model and enhance calculation efficiency, a comparative runtime analysis was conducted. Under identical simulation parameters (Table 1) and computing configurations (Table 6), the total runtime for the direct integration model was 20,309.5863 s, whereas the Fresnel Simplified Model required only 32.4756 s. This corresponds to an efficiency improvement by a factor of approximately 625.38. These results effectively demonstrate that the simplified model achieves an optimal balance between high computational accuracy and simulation efficiency.

#### 3.3.3. Simplified Model Compensation Analysis

Although the Fresnel Simplified Model achieves high accuracy regarding key performance metrics, such as the beam shape and phase distribution of the normalized pattern, it inevitably exhibits a systematic deviation in absolute gain. This fixed deviation primarily originates from two sources:

1. To derive an analytical solution, the Fresnel approximation treats the slowly varying amplitude term as a constant. This simplification neglects subtle variations relative to the integration variable and overestimates the current contribution at the reflector edges. While this has a negligible impact on the beam shape, it induces a constant-level shift in the absolute gain.

2. The truncation of higher-order terms in the Taylor series expansion exerts a slight influence on the coherent superposition at the main lobe peak. This results in discrepancies in the far-field peak field strength compared to that of the direct integration model.

To achieve precise calibration of the absolute gain for the simplified model, while accounting for the minor differences in energy response between the main lobe and sidelobe regions under the Fresnel approximation, this paper proposes a general partitioned calibration compensation method.

Regarding the main lobe of the antenna pattern, the absolute gain of the direct integration model within the primary −3 dB region of interest serves as the benchmark. Based on the RMSE criterion, the absolute gain deviation $\Delta G_{Main}$ between the simplified model and the benchmark is calculated under various error conditions. Statistical analysis reveals that the standard deviation of $\Delta G_{Main}$ with respect to error variations is 0.0481 dB. This indicates that $\Delta G_{Main}$ does not vary significantly with perturbations in structural error. Consequently, the absolute gain deviation observed in the ideal state can be treated as a fixed correction factor $K_{Main}$ for the main lobe range.

For the near sidelobe regions located away from the main axis, the absolute gain error introduced by the Fresnel approximation may differ slightly from that of the main lobe. To achieve optimal global compensation, the peaks of the first *N* sidelobes are selected as references. Similarly, based on the minimum RMSE criterion, the absolute gain deviation $\Delta G_{Side}$ is obtained via a weighted average according to the $\Delta G_{Main}$ across error variations, which is 0.0015 dB. This result confirms that the deviation indicates insensitivity to perturbations in structural error. Therefore, a fixed correction factor $K_{Main}$ for the sidelobe region can be established.

To combine the main lobe and sidelobe correction effects and systematically compensate for the simplified model’s absolute gain deviations, a weight function W(θ) is designed with the pattern’s first nulls $\pm \theta_{0}$ as boundaries, enabling a smooth transition between main lobe and sidelobe compensation. The weight function is defined as shown in (33).

(33) $$W\left(\theta \right)=\frac{1}{2}\left[1+\tanh\left(\frac{\left|\theta \right|-\theta_{0}}{\tau }\right)\right]$$

The aforementioned weight function takes W(θ)=0 in the main lobe region, W(θ)=1 in the sidelobe region, and realizes a smooth transition from 0 to 1 near the first nulls $\pm \theta_{0}$. Here, τ serves as a shape factor governing the steepness of the main lobe and sidelobe smooth transition region.

Based on the above mentioned weight function W(θ), the main lobe’s correction factor $K_{Main}$, and the sidelobe’s correction factor $K_{Side}$, the compensated simplified model is obtained:

(34) $$\begin{array}{c} F_{r\_comp}^{\prime }\left(\theta \right)=F_{r}^{\prime }\left(\theta \right)-\left[\left(1-W(\theta )\right)\;\cdot \;K_{Main}+W\left(\theta \right)\;\cdot \;K_{Side}\right] \end{array}$$

**Figure 12:**
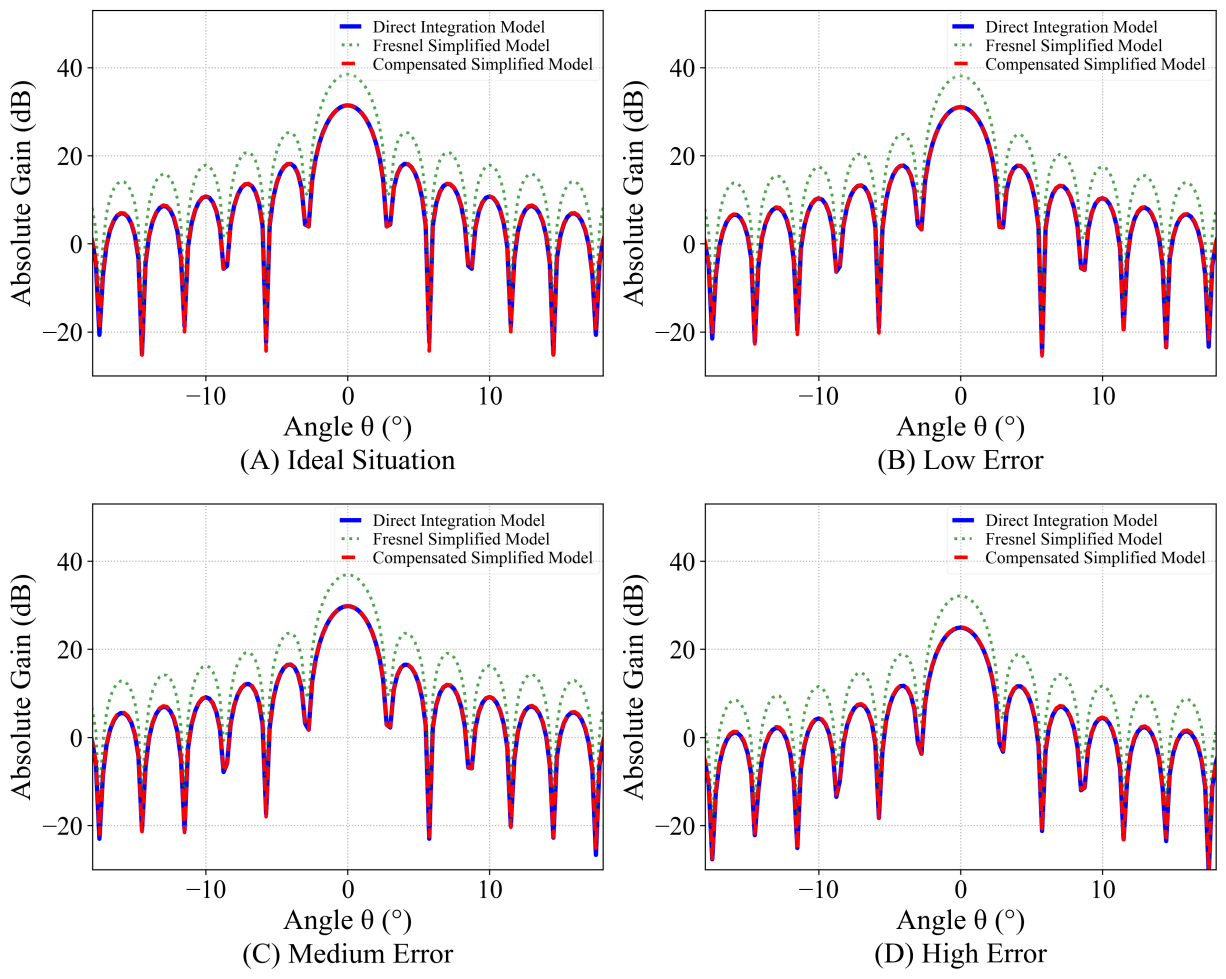
Absolute gain compensation effects.

Fundamentally, the magnitude of the compensation factor is determined by the geometric parameters of the parabolic cylindrical antenna, such as the focal ratio. Specifically, as the f/D ratio decreases, the curvature of the reflector increases, leading to a more pronounced absolute gain deviation introduced by the Fresnel approximation. Based on the antenna parameters configured in this study and the proposed partitioned calibration method, simulation calculations yield $K_{Main}$ = 7.0888 dB and $K_{Side}$ = 7.1325 dB. The difference between these two compensation factors is merely 0.0437 dB. This discrepancy is negligible regarding the absolute gain of the antenna sidelobes, a global compensation factor $K=K_{Main}=K_{Side}$ = 7.0888 dB is adopted to simplify the analysis process. The compensation results are illustrated in Figure 12.

To quantify compensation efficacy and the accuracy of the compensated simplified model, absolute gain compensation results for the local main lobe region and first five sidelobes on either side of the main lobe across all error conditions are tabulated in Table 7.

The results demonstrate that the compensated Fresnel Simplified Model accurately matches the radiation pattern energy of the direct integration model across all error levels. This calibration corrects the systematic increase in absolute gain caused by the Fresnel approximation. The proposed method effectively eliminates the systematic gain deviation inherent to the approximation, while preserving the simplified model’s key advantages of high computational efficiency and high waveform fidelity.

## 4. Fresnel Simplified Model Application Condition Analysis

**Figure 13:**
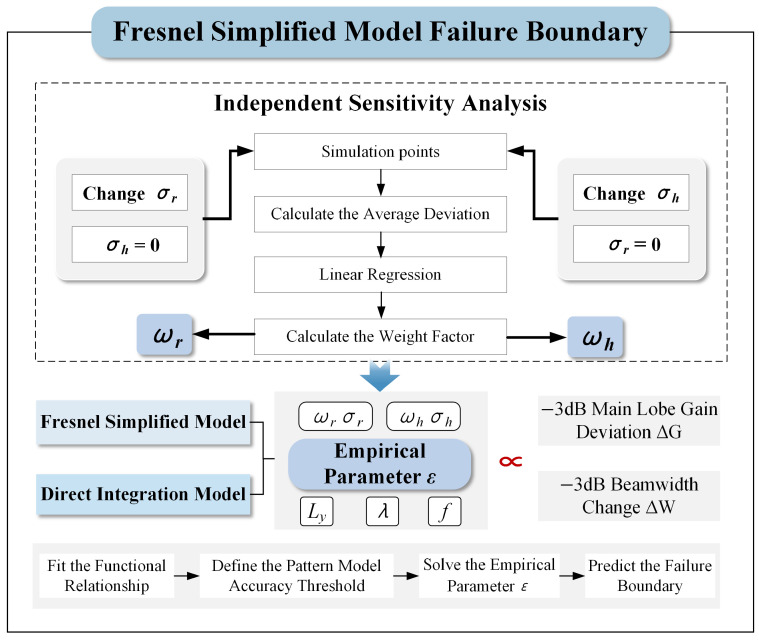
The failure boundary simulation process of the simplified Model.

Building upon the model established in the preceding sections, this chapter defines empirical parameter ε to quantify the relationship between multi-source structural errors and the prediction accuracy of the simplified model. Furthermore, the validity limits of the Fresnel Simplified Model are investigated through simulation. The detailed simulation workflow is illustrated in Figure 13.

The Fresnel approximation requires the paraxial condition. Specifically, for small reflector error, moderate antenna size, and large focal lengths, the contribution of high-order terms in the phase Taylor expansion to the integral is negligible, and the quadratic approximation suffices to accurately capture key antenna pattern information. The physical constraint of this approximation is that the phase variation induced by the dominant discarded high-order terms must be far smaller than one electromagnetic wave phase period [45]:

(35) $$\max\left(k\;\cdot \;\frac{{\left(y-y_{n}^{\prime }\right)}^{4}}{8D_{mn}^{3}}\right)\ll 2\pi$$

The above equation shows that the validity of the Fresnel approximation depends strongly on error magnitude, antenna size, wavelength, and focal length. For large error magnitudes, increased antenna size, shorter wavelength, or reduced focal length, the model deviates from the paraxial approximation condition and becomes invalid. To quantitatively assess the failure boundaries of the compensated simplified model under different structural errors in subsequent simulations, an empirical parameter ε incorporating the aforementioned key factors is defined as shown in (36).

(36) $$\varepsilon =\frac{\sqrt{{\left(\omega_{r}\sigma_{r}\right)}^{2}+{\left(\omega_{h}\sigma_{h}\right)}^{2}}\;\cdot \;L_{y}}{\lambda \;\cdot \;f}$$

where $\sigma_{r}$ and $\sigma_{h}$ respectively represent the structural errors of the linear array feed and the parabolic cylindrical reflector, $\omega_{r}$ and $\omega_{h}$ respectively represent the error weight relationship of the linear array feed and the parabolic cylindrical reflector. The control variable method is employed to conduct a sensitivity analysis on the two error types, yielding the corresponding weighting ratios. Based on these ratios, simulations were performed to analyze both the antenna radiation performance and the simplified model’s prediction accuracy. The results indicate that reflector structural errors are the dominant factor influencing the antenna’s radiation performance. Consequently, in antenna design and manufacturing, stricter control of reflector accuracy is essential to ensure the stability and reliability of overall performance. In contrast, for the simplified model, feed structural errors have a greater impact on prediction accuracy. This is because variations in the feed structure compromise the paraxial condition required for the Fresnel approximation, making the model’s accuracy more sensitive to feed errors.

**Figure 14:**
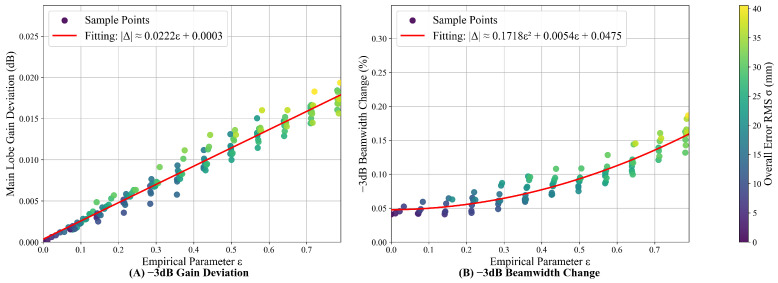
The fitting relationship between the main lobe prediction deviation and ε.

To clarify the validity limit and failure boundary of the Fresnel Simplified Model, a quantitative relationship between model prediction accuracy and structural errors is established using an empirical parameter ε. First, the weighting ratio between the two error types in the combined error is determined by calculating the sensitivities of the feed and reflector errors to the pattern performance of the parabolic cylindrical antenna. Based on these weighting ratios, multiple sets of error data are constructed. The main lobe gain prediction deviation ΔG and the −3 dB beamwidth variation ΔW of the Fresnel Simplified Model are calculated. These metrics are then individually fitted against the empirical parameter ε, with the simulation results illustrated in Figure 14. Based on these fitting results, combined with the specific physical parameter of the antenna and the accuracy requirements of the model, the critical value of the empirical parameter and the allowable structural error thresholds for a specific accuracy level are derived. This threshold explicitly defines the effective validity boundary of the model. In practical engineering applications, if the expected structural errors of an antenna exceed this threshold, the direct integration model should be selected for pattern performance evaluation to ensure the authenticity and reliability of the results.

Figure 14 shows that the model’s −3 dB main lobe gain prediction deviation has a linear fit ($R^{2}=$ 0.9684, RMSE= 0.0009 dB) with the empirical parameter ε, while the −3 dB beamwidth variation approximately shows a quadratic relationship ($R^{2}=$ 0.8875, RMSE= 0.0123%). Fitting results are tabulated in Table 8.

Aforementioned results show that the empirical parameter ε correlates positively with main lobe gain prediction deviation but with an extremely small slope, confirming the model’s exceptional stability in gain prediction. Using 0.2 dB as the gain prediction accuracy threshold, the equivalent structural error derived from ε inversion is far beyond the antenna wavelength λ and engineering tolerances, further validating the compensated simplified model’s practical effectiveness. On the other hand, the relationship between the variation in the −3 dB beamwidth and the empirical parameter ε exhibits a quadratic polynomial correlation. By establishing a 10% threshold for beamwidth variation, the derived equivalent structural error is found to significantly exceed the antenna wavelength. This margin far surpasses standard engineering precision requirements. Based on this, the Fresnel Simplified Model demonstrates an extensive tolerance for structural errors in practical engineering applications, enabling the efficient evaluation of the impact of combined structural errors on the performance of parabolic cylindrical antennas.

## 5. Conclusions and Prospects

In this paper, a radiation pattern modeling method for linear array-fed parabolic cylindrical antennas is established. This method comprehensively accounts for structural errors in both the feed and the reflector, facilitating efficient and high-precision calculations. Verified via FEKO simulations, the direct integration pattern model serves as a robust benchmark for high-precision modeling. Furthermore, the simplified model derived from the Fresnel approximation demonstrates an efficiency improvement by a factor of approximately 625 while maintaining exceptional fidelity in beam morphology (with a normalized main lobe amplitude RMSE of less than 0.01 dB and phase RMSE of less than 0.03°). To address global absolute gain compensation, a general partitioned calibration strategy is proposed. Given that the difference between the absolute gain compensation factors for the main lobe and sidelobes is less than 0.05 dB in this study, a global compensation factor of *K* = 7.0888 dB is determined, which effectively eliminates systematic deviations. Simulation results indicate that the Fresnel Simplified Model exhibits robust error tolerance while meeting specific accuracy requirements. It can effectively replace the direct integration model across various error environments, meeting the demands of high-efficiency and high-precision applications. These findings provide a theoretical foundation for the tolerance design of parabolic cylindrical antennas and serve as an efficient tool for pattern prediction, offering significant engineering value for enhancing the performance robustness of antenna systems.

Future research will focus on two primary directions:

1. Incorporating the analysis of complex mechanisms (such as dielectric loss, mutual coupling, multi-polarization, and large-angle scanning) into the modeling process to enhance the model’s universality and precision.

2. Leveraging the established pattern model to conduct reverse error inversion during practical applications. This will enable the precise localization and identification of antenna structural errors, thereby elevating the intelligence level of antenna operation, maintenance, and performance optimization.

## Figures and Tables

**Table 1:** Quantitative comparison of metrics between the Direct Integration Model and FEKO simulation.

Scanning Angle	Metric	Direct Integration Model	FEKO Simulation	Deviation
0°	−3 dB Beamwidth (°)	2.5334	2.5177	0.0158
	Beam Pointing (°)	0	0	0
	−3 dB Gain RMSE (dB)	-	-	0.0211
5°	−3 dB Beamwidth (°)	2.5432	2.5298	0.0133
	Beam Pointing (°)	5	5	0
	−3 dB Gain RMSE (dB)	-	-	0.1886
10°	−3 dB Beamwidth (°)	2.5722	2.6282	−0.0559
	Beam Pointing (°)	10	10	0
	−3 dB Gain RMSE (dB)	-	-	0.0869

**Table 2:** Simulation parameters setting of a linear array-fed parabolic cylindrical antenna.

Parameter Value	Parameter Description
*f* = 1.6 m	Parabolic cylindrical focal length
$W_{x}$ = 4 m	Parabolic cylindrical aperture
$L_{y}$ = 10 m	Parabolic cylindrical length along the focal line
M=100	Total number of discrete elements in the direction perpendicular to the focal line
N=32	Total number of linear feeding elements
*d* = 0.15 m	Spacing between adjacent feeding elements
*f* = 1.25 GHz	Frequency
$\begin{array}{c} q=1 \end{array}$	Beamwidth factor
λ = 0.24 m	Wavelength
$\begin{array}{c} {\hat{e}}_{p}=\left(1,0,0\right) \end{array}$	Horizontal polarization

**Table 3:** Setting of simulation error level parameters.

Error Level	Feed Structure Error *σ*(*m*)	Feed Relative Error	Reflector Structure Error *σ*(*m*)	Reflector Relative Error
Low	0.0048	0.02*λ*	0.0060	0.025*λ*
Medium	0.0096	0.04*λ*	0.0120	0.05*λ*
High	0.0192	0.08*λ*	0.0240	0.10*λ*

**Table 4:** Statistical results of normalized amplitude deviation within the main lobe between the Direct Integration Model and the Fresnel Simplified Model.

Error Level	Main Lobe Range	Normalized Amplitude RMSE (dB)	Max Normalized Amplitude Deviation (dB)
Ideal	−3 dB	0.0015	0.0026
	−5 dB	0.0018	0.0029
	−10 dB	0.0018	0.0029
Low	−3 dB	0.0019	0.0041
	−5 dB	0.0022	0.0047
	−10 dB	0.0024	0.0047
Medium	−3 dB	0.0024	0.0053
	−5 dB	0.0028	0.0060
	−10 dB	0.0033	0.0061
High	−3 dB	0.0025	0.0060
	−5 dB	0.0030	0.0070
	−10 dB	0.0042	0.0092

**Table 5:** Statistical results of main lobe phase deviation between the Direct Integration Model and the Fresnel Simplified Model.

Error Level	Phase RMSE (°)	Max Phase Deviation (°)
Ideal	0.0122	0.0183
Low	0.0138	0.0211
Medium	0.0173	0.0284
High	0.0286	0.0488

**Table 6:** Computing environment configurations.

Configuration Item	Parameters
Processor	Intel(R) Core(TM) i7-1056G7 CPU@1.30 GHz, 1498 MHz, 4 Cores 8 Threads
Operating System	Windows 10 64-bit
Simulation Environment	Python 3.11
RAM	8.0 GB
Disk Capacity	954 GB

**Table 7:** Statistical results of absolute gain compensation for the Fresnel Simplified Model under different errors.

Error Level	Main Lobe −3 dB Absolute Gain RMSE (dB)	Absolute Gain RMSE of First 5 Sidelobes (dB)
Ideal	0.0065	0.1062
Low	0.0074	0.1117
Medium	0.0259	0.1268
High	0.1162	0.2153

**Table 8:** The fitting relationship between ε and the main lobe prediction deviation.

Parameter	−3 dB Main Lobe Gain Prediction Deviation	−3 dB Beamwidth Variation
$R^{2}$	0.9684	0.8875
RMSE	0.0009 dB	0.0123%
Fitting relationship	$\left|\Delta G\right|\approx$ 0.0222*ε* + 0.0003	$\left|\Delta W\right|\approx$ 0.1718*ε*^2^ + 0.0054*ε* + 0.0475

## Data Availability

The author declare no new data were created. The relevant parameters are contained within the article.
